# Effect of Ti Addition on the Microstructure and Mechanical Properties of L-PBF-Fabricated CoCrNi Medium-Entropy Alloys

**DOI:** 10.3390/ma19214651

**Published:** 2026-10-31

**Authors:** Zhongqiang Liu, Shuya Liang, Guan Xu, Siyuan Peng

**Affiliations:** 1 Well Technology Company Limited, Foshan 528225, China; zhqliurp@gmail.com (Z.L.); liangshuya@zhwt.com.cn (S.L.); xg@zhwt.com.cn (G.X.); 2 School of Materials Science and Engineering, Xiamen University of Technology, Xiamen 361024, China

**Keywords:** CoCrNi medium-entropy alloy, titanium alloying, laser powder bed fusion, additive manufacturing

## Abstract

Laser powder bed fusion (L-PBF) delivers significant advantages for manufacturing medium-/high-entropy alloys (MEAs/HEAs), owing to its near-net-shape manufacturing and the ability to fabricate complex geometries. In this paper, a series of (CoCrNi)_100−x_Ti_x_ (x = 0, 1, 2, 4 wt.%) MEAs were successfully fabricated by L-PBF technology, and the effect of Ti addition on the microstructure and mechanical properties was systematically investigated. The results show that L-PBF CoCrNi-4wt.%Ti alloy processed with a laser power of 275 W and the scanning speed of 1000 mm/s delivered a high yield strength of 777.5 MPa and ultimate tensile strength of 1054.5 MPa, along with a ductility of 20.8%. Compared with the L-PBF CoCrNi alloy, the CoCrNi-4 wt.%Ti alloy shows an increase in yield strength from 684.8 to 777.5 MPa and in ultimate tensile strength from 961.1 to 1054.5 MPa, accompanied by a decrease in uniform elongation from 27.8% to 20.8% (a reduction of seven percentage points, approximately 25% relative). The present work offers a promising pathway to improve the performance of additively manufactured medium-entropy alloys.

## 1. Introduction

CoCrNi medium-entropy alloys (MEAs) consist of three main elements—Co, Cr, and Ni—and have attracted significant attention due to their excellent balance of strength and ductility, thermal stability, and radiation resistance at low temperatures [1,2]; however, their room-temperature strength falls short of the stringent requirements in fields such as aerospace.

To address this, researchers have primarily sought breakthroughs through process optimization and alloying: on the one hand, advanced processes such as spark plasma sintering (SPS) can enhance strength by refining the grain structure; on the other hand, lattice distortion induced by the addition of elements with large atomic radii (such as W) can achieve solid solution strengthening while maintaining ductility [3,4,5,6,7]. Laser powder bed fusion (L-PBF) technology enables the fabrication of complex metal components with high precision and excellent mechanical properties. The rapid, non-equilibrium solidification inherent to L-PBF promotes fine-grained microstructures and induces a high density of dislocations, often forming cellular substructures [8]. These features contribute significantly to dislocation strengthening and Hall-Petch-type grain boundary strengthening, thereby enhancing yield strength [9]. However, L-PBF-fabricated CoCrNi alloys still face challenges in achieving optimal strength-ductility synergy. Recent studies have demonstrated that the incorporation of second-phase particles, such as ceramics, can significantly enhance the strength of the alloys through mechanisms such as Orowan strengthening and dislocation pinning [10,11,12].

The incorporation of hard ceramic reinforcements—such as TiO_2_, TiC, TiN, Y_2_O_3_, and B_4_C—can substantially enhance the strength, corrosion resistance, and wear resistance of the CoCrNi alloys [4,8,10,11,12,13,14,15,16,17,18,19,20,21,22]. For instance, Tang et al. [8] reported that CoCrNi composites reinforced with 1–2 wt% TiN nanoparticles were fabricated by L-PBF, achieving a remarkable synergy of strength and ductility. The addition of 1 wt% TiN increased the yield strength and ultimate tensile strength from 694.5 MPa to 955 MPa–806 MPa and 1084 MPa, respectively, while the fracture elongation remained comparable (33% to 33.5%). Previous studies have examined how individual element additions (Nb, W, Fe, Mo, Cu) influence the mechanical characteristics of CoCrNi medium-entropy alloys. Titanium (Ti), exhibiting a hexagonal close-packed (HCP) structure at room temperature, tends to form body-centered cubic (BCC) phases in multicomponent alloys. Ti is commonly incorporated to improve strength and corrosion resistance through solid solution or precipitation strengthening mechanisms, enabling the design of low-density, high-strength alloy systems [23]. Khan et al. [24] investigated the effectiveness of solute-driven constitutional supercooling for grain refinement in CoCrNi-based MEAs processed by L-PBF, using Ti as a model solute. Although thermodynamic calculations predicted a strong growth-restricting effect of Ti, the addition of 3 at% Ti did not result in grain refinement. However, the mechanical properties of these Ti-modified CoCrNi alloys were not evaluated, leaving the structure–property relationship underexplored. Recently, Ji et al. [13] reported a series of (CoCrNi)_100−x_Ti_x_ (x = 0, 1, 3, 5, 8) MEAs fabricated by L-PBF and systematically investigated the effect of Ti content on their microstructural evolution and mechanical properties. The addition of Ti promoted the in situ formation of TiO_2_ nanoparticles, which exhibited pronounced segregation along the cellular structures. However, owing to equipment limitations (the maximum output power of the laser was 200 W), the process–microstructure–mechanical property relationships were not systematically investigated.

In this work, a series of (CoCrNi)_100−x_Ti_x_ (x = 0, 1, 2, 4 wt.%) MEAs were successfully fabricated by L-PBF technology. The influence of Ti addition on the printing parameters, microstructure, and mechanical properties of the L-PBF-ed CoCrNi was systematically studied. The study offers a promising pathway to improve the performance of additively manufactured medium-entropy alloys.

## 2. Materials and Methods

### 2.1. Thermodynamic Calculations

**Figure 1:**
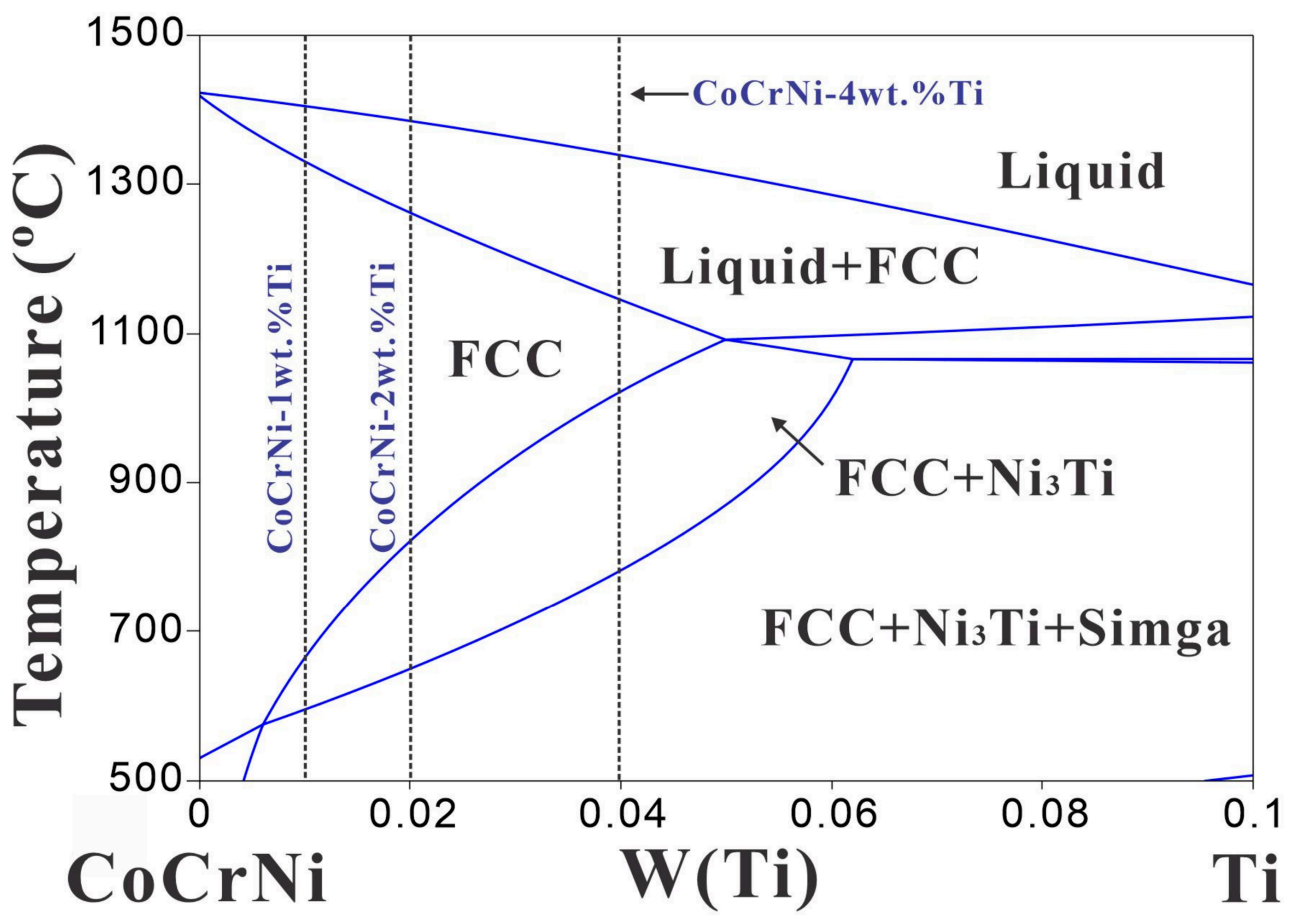
Thermodynamic predictions for the pseudo-binary equilibrium phase diagram of (CoCrNi)*_100-_*_x_-(Ti)_x_ alloys.

Thermodynamic calculations were performed using Pandat 2025 with the PanHEA2024 database (CompuTherm LLC, Middleton, WI, USA) to guide the compositional design. Figure 1 shows the pseudo-binary (CoCrNi)_100-*x*_-(Ti)*_x_* (wt.%) equilibrium phase diagram from CoCrNi to Ti with phase field labels. Thermodynamic calculations predicted that the topologically close-packed (TCP) sigma (σ) phase would readily form when the Ti content exceeded 5 wt.%. However, the sigma phase is very hard and brittle and can significantly affect the mechanical properties [25]. Based on the results of thermodynamic calculations, four representative (CoCrNi)_100-x_-(Ti)_x_ (x = 0, 1, 2, 4 wt.%) alloys were designed and labeled CoCrNi, CoCrNi-1wt.%Ti, CoCrNi-2wt.%Ti and CoCrNi-4wt.%Ti, respectively.

### 2.2. L-PBF Processing

This study used spherical CoCrNi pre-alloyed powders (particle size 15–53 μm, Jiangsu VILORY New Materials Technology Co., Ltd., Xuzhou, Jiangsu, China) prepared by the gas atomization method and irregularly shaped Ti particles (99.99%, cas: 7440-32-6, Aladdin, Shanghai, China) as raw materials. The particle size distribution (PSD) was determined by laser diffraction (Mastersizer MS3000E, Malvern Panalytical, UK). To obtain a powder mixture with uniform composition, CoCrNi powder and Ti powder with different mass fractions were first mixed using an ultrasonic vibration disperser (JXSF-G2, Shanghai Jingxin Industrial Development Co., Ltd., Shanghai, China) at a power of 120 W and a frequency = 30 KHZ for 5–7 cycles, with each mixing cycle lasting 2–2.5 min, and then mechanically mixed in a V-type mixer at 24 rpm for 12 h, to ensure uniform Ti distribution [26].

The samples were fabricated using a WXL-120 laser powder bed fusion system (Wuxinglong, Technology Co., Ltd. Xiamen, China) with a laser beam size of 70 μm. To ensure uniform powder deposition during printing and effectively reduce the impact of residual stress on print quality, the build plate was leveled and preheated to 100 °C. The entire process was conducted under a continuous flow of high-purity nitrogen as the protective atmosphere, with the oxygen content in the build chamber maintained below 300 ppm. To determine the optimal parameters, the layer thickness (*t*) and hatch spacing (*h*) were kept at 0.04 mm and 0.05 mm, respectively. The volumetric energy density (VED = P/vht) was calculated to explore the effects of processing parameters on the printing quality and microstructural evolution. (Table S1 in the Supplementary Materials shows the list of processing parameters for L-PBF fabrication). Based on the Archimedes principle, the density of the samples was measured. The relationships between the relative densities of the L-PBF CoCrNi, CoCrNi-1wt.%Ti, CoCrNi-2 wt.%Ti, CoCrNi-4wt.%Ti and the volume energy density are shown in Table S2. In order to investigate the influence of Ti addition, near-fully dense samples were successfully fabricated with the optimized processing condition of 275 W laser power and 1000 mm/s scanning speed. The scan strategy was a chessboard pattern with a rotation of 67° between layers.

### 2.3. Microstructure and Mechanical Properties Characterization

The phase identification of the raw powders and as-fabricated samples was performed via X-ray diffraction (XRD, SmartLab 3 kW, Rigaku Corporation, Tokyo, Japan) with Cu Kα radiation scanning from 20 to 90°; the step size was 0.010°, and the counting time was 1 s. The microstructural characteristics of the specimens were examined using an optical microscope (SOPTOP, CX40M, Ningbo Shunyu Instruments Co., Ltd., Yuyao, Zhejiang, China) and field emission scanning electron microscopy (Zeiss Sigma 500, Oberkochen, Germany) equipped with energy-dispersive X-ray spectroscopy (EDS). An electron backscatter diffraction (EBSD, Oxford Instruments Group, High Wycombe, UK) system was used to quantitatively evaluate the grain size and crystallographic texture. Prior to EBSD testing, the samples were polished using a 40 nm colloidal silica suspension for 30–50 min to improve surface quality and release residual stress. EBSD measurements were carried out at a step size of 0.04 μm. EBSD data were analyzed using Channel 5 data analysis software.

**Figure 2:**
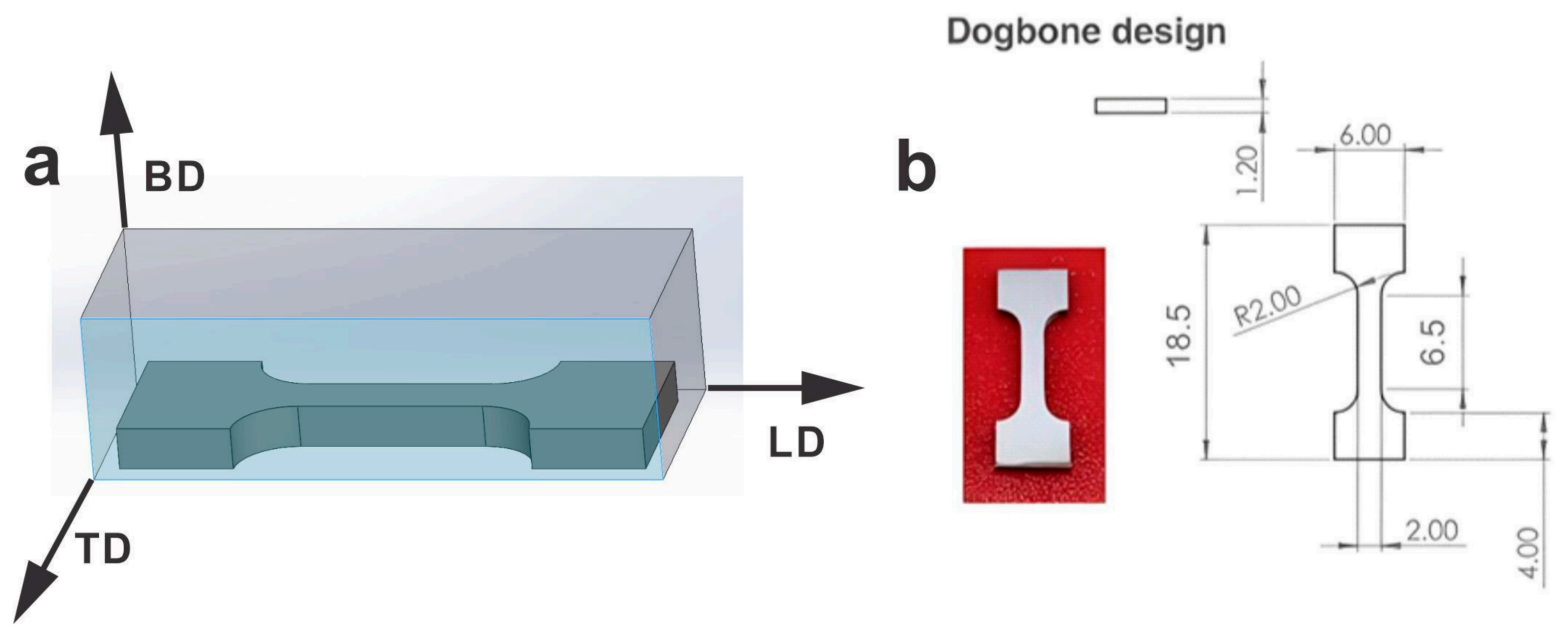
(**a**) Schematic of the bidirectional and cross-hatched laser scan strategy. BD: build direction, LD: longitudinal direction, TD: transverse direction. (**b**) Schematic diagram of tensile test specimen dimensions.

Vickers hardness testing was performed using an HVS-50 digital Vickers hardness tester with a load of 200 g and a hold time of 10 s (Figure S2 in the Supplementary Materials shows the Vickers hardness results of L-PBF CoCrNi, CoCrNi-1wt.%Ti, CoCrNi-2wt.%Ti and CoCrNi-4wt.%Ti alloys). Ten measurements were taken on each specimen, and the average value was calculated. The dog-bone-shaped specimens with a gauge section of 6.5 mm × 2 mm × 1.2 mm (length × width × thickness) were tested at room temperature using an MTS Alliance RT30 universal testing machine at a strain rate of 2 × 10^−4^ s^−1^; yield strength was determined using the 0.2% strain offset method; and three independent specimens were tested for each condition under identical specimen orientation and testing conditions, as shown in Figure 2. A contact extensometer was used for precise strain measurement. Before the test, the sample surfaces were polished with 1200-grit SiC paper. The tests were repeated three times to minimize experimental errors.

## 3. Results and Analysis

### 3.1. Microstructure and Phase Analysis

#### 3.1.1. SEM Image

**Figure 3:**
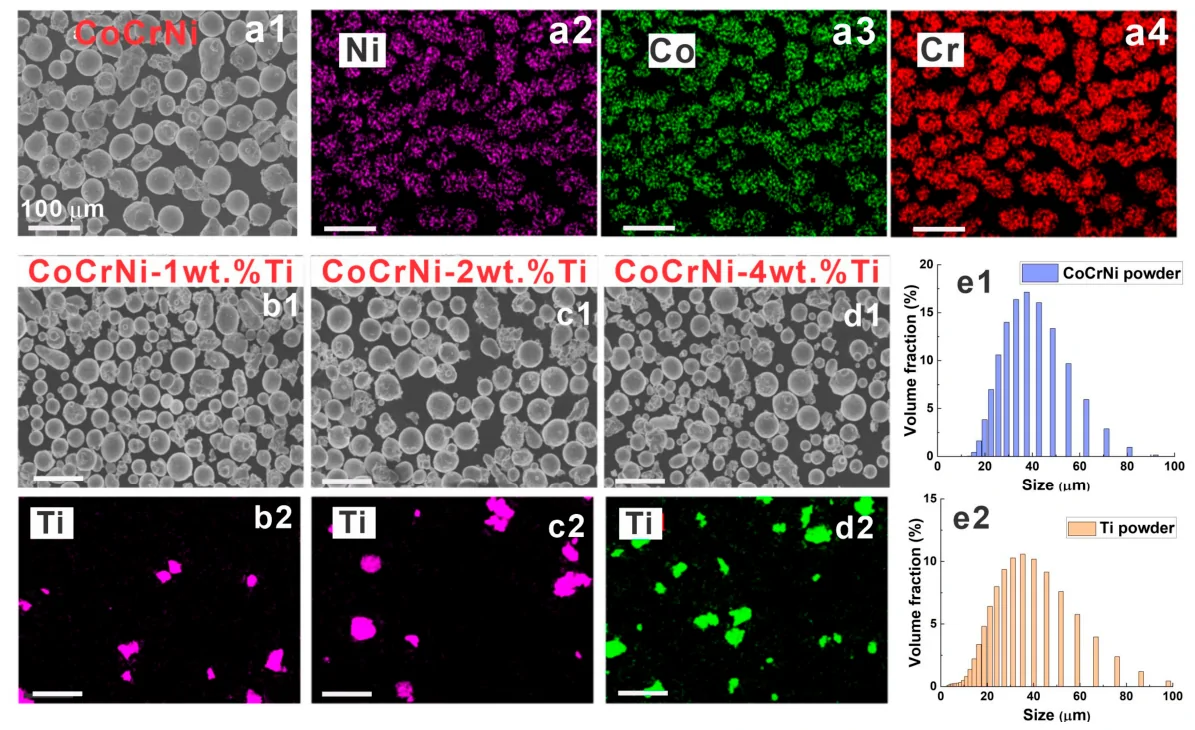
SEM Images of (CoCrNi)_100−x_Ti_x_ (x = 0, 1, 2, 4 wt.%) MEAs: (**a1**–**a4**) SEM image of pure CoCrNi powders; (**b1**,**b2**) SEM image of CoCrNi-1wt%Ti powders; (**c1**,**c2**) SEM image of CoCrNi-2wt% Ti powders; (**d1**,**d2**) SEM image of CoCrNi-4wt%Ti powders; particle size distribution of (**e1**) CoCrNi powders and (**e2**) Ti powders.

Figure 3 shows SEM images of CoCrNi powders with different Ti contents. The irregularly shaped particles shown in the figure are trace amounts of Ti powder added to the CoCrNi powder in Figure 3b. A comparison of Ti contents x = 0, 1, 2, and 4 wt.% reveals that the distribution of Ti atoms is uniform, thereby preventing excessive variations in the local mechanical properties of the prepared composite material. The particle size distribution (PSD) exhibited a D_10_ = 23.8 μm, D_50_= 36.9 μm, D_90_ = 57.0 μm, and D_10_ = 18.7 μm, D_50_= 35.9 μm, D_90_ = 64.1 μm for CoCrNi and Ti powders, respectively, as shown in Figure 3(e1,e2).

#### 3.1.2. XRD Phase Analysis

**Figure 4:**
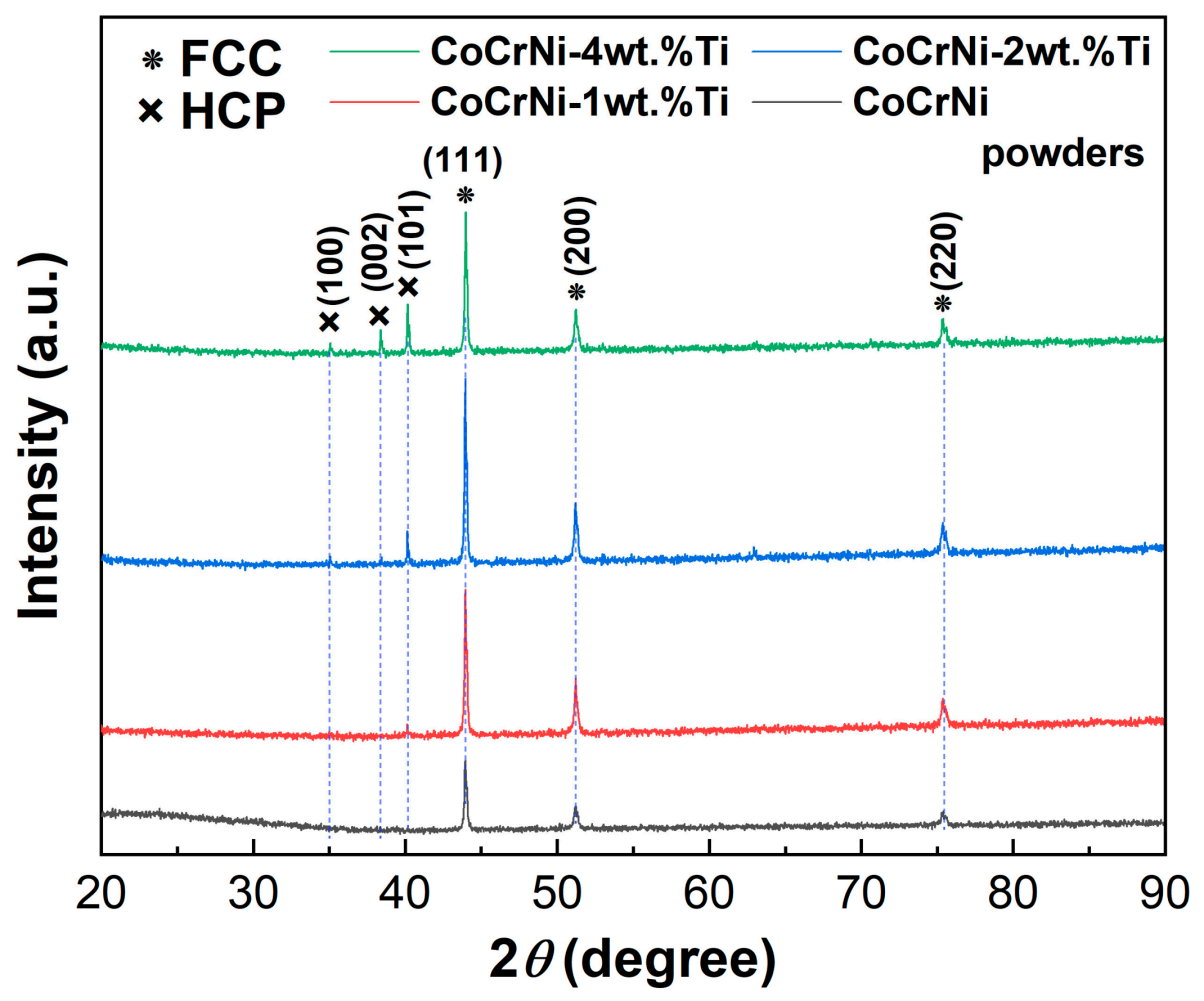
XRD patterns of the CoCrNi, CoCrNi-1wt%Ti, CoCrNi-2wt%Ti and CoCrNi-4wt%Ti powders.

Figure 4 shows the XRD patterns of (CoCrNi)_100−x_Ti_x_ (x = 0, 1, 2, 4 wt.%) MEAs. The XRD pattern of pure CoCrNi powders showed only an FCC structure (marked with an asterisk *), corresponding to the face-centered cubic lattice of a typical CoCrNi solid solution. The diffraction peaks near 2θ = 44°, 51°, and 75 ° correspond to FCC planes (such as (111), (200), and (220)). The XRD patterns of CoCrNi-Ti composite powders with different Ti contents showed both FCC phase and HCP-Ti phase (JCPDS #00-005-0682) peaks (marked with an asterisk ×). With increasing Ti content, the diffraction peaks (100), (002), and (101) of the HCP-Ti phase become more pronounced.

**Figure 5:**
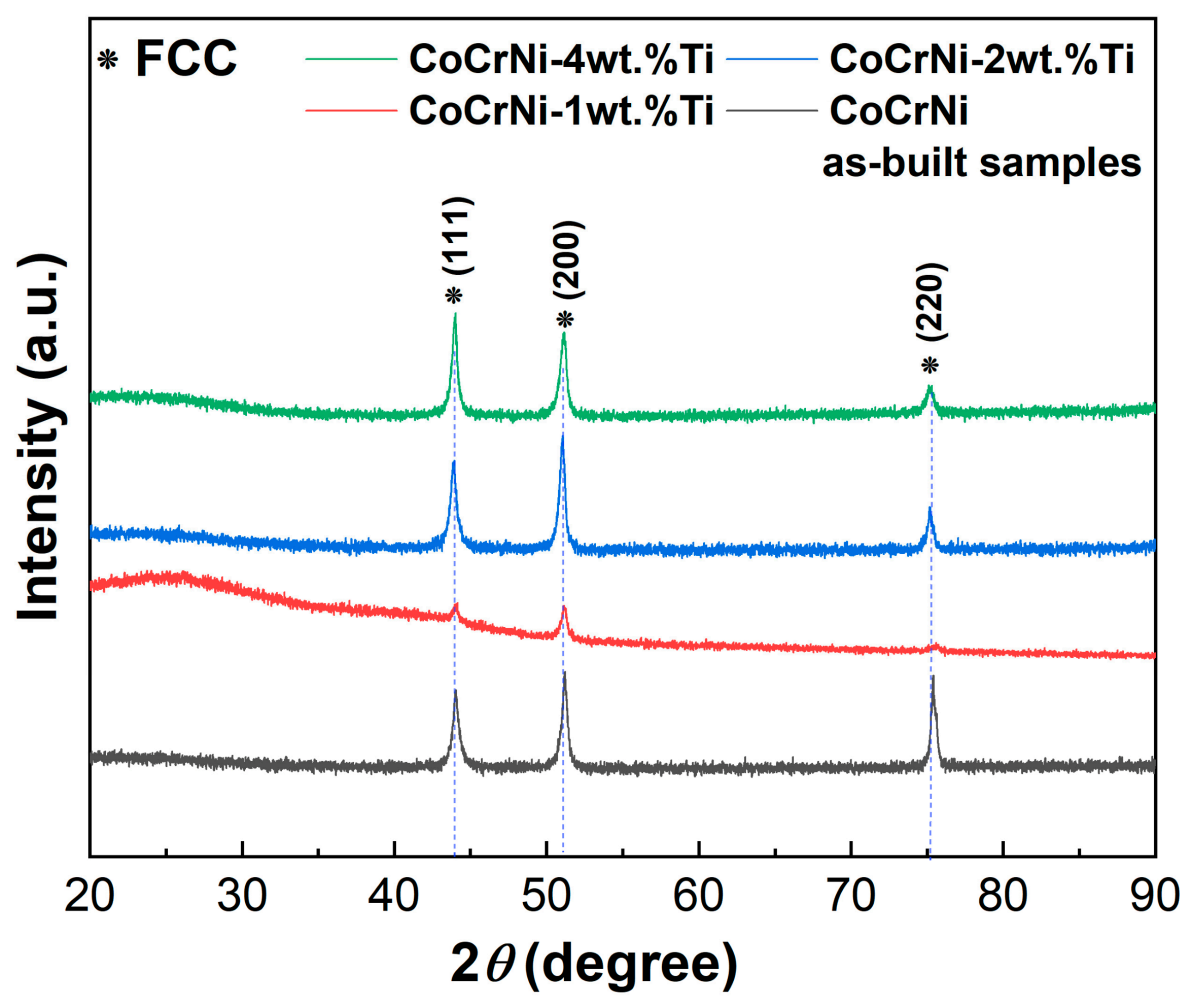
XRD patterns of the as-built L-PBF CoCrNi, CoCrNi-1wt%Ti, CoCrNi-2wt%Ti and CoCrNi-4wt%Ti samples.

Figure 5 shows the XRD patterns of as-built CoCrNi, CoCrNi-1wt%Ti, CoCrNi-2wt%Ti and CoCrNi-4wt%Ti samples. Due to the relatively low Ti content, there were no obvious HCP-Ti phase peaks in the XRD patterns of L-PBF CoCrNi-1wt.%Ti, CoCrNi-2wt.%Ti, and CoCrNi-4wt%Ti samples. The XRD results show that the FCC structure was detected in the as-built CoCrNi, CoCrNi-1wt%Ti, CoCrNi-2wt%Ti, and CoCrNi-4wt%Ti samples.

#### 3.1.3. Microstructural Evolution

**Figure 6:**
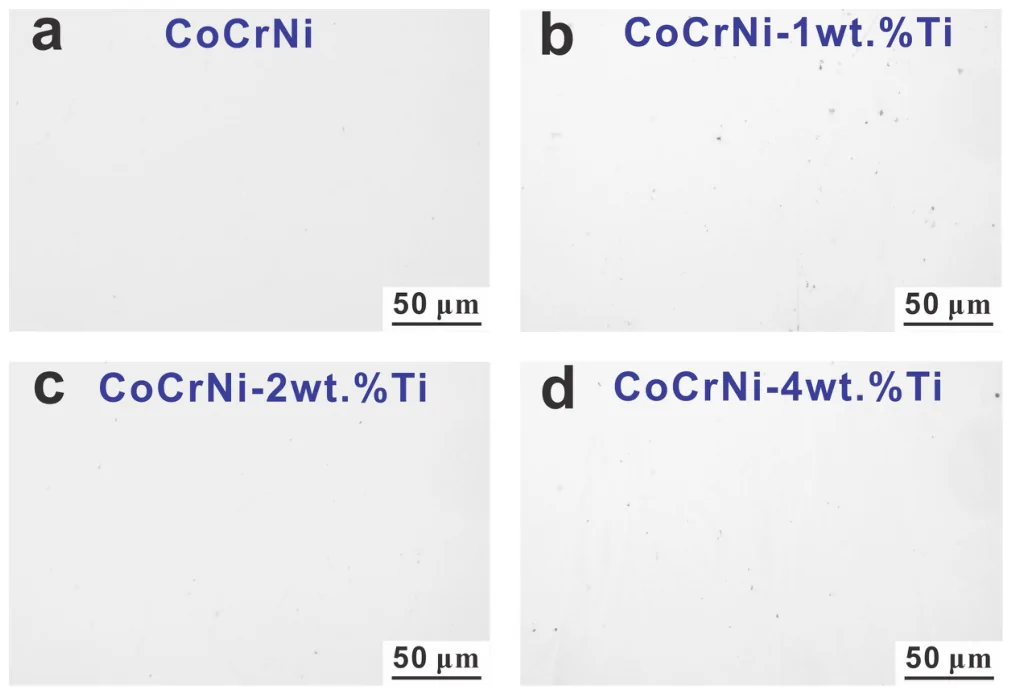
Optical microscopy images of L-PBF (**a**) CoCrNi, (**b**) CoCrNi-1wt.%Ti, (**c**) CoCrNi-2wt.%Ti and (**d**) CoCrNi-4wt.%Ti alloys at the build direction.

Figure 6 presents optical microscopy (OM) images showing defect distribution in the CoCrNi, CoCrNi-1wt.%Ti, CoCrNi-2wt.%Ti and CoCrNi-4wt.%Ti alloys. Lack-of-fusion defects were rarely observed, while a small number of gas-trapped pores were present in the CoCrNi-1wt.%Ti sample.

**Figure 7:**
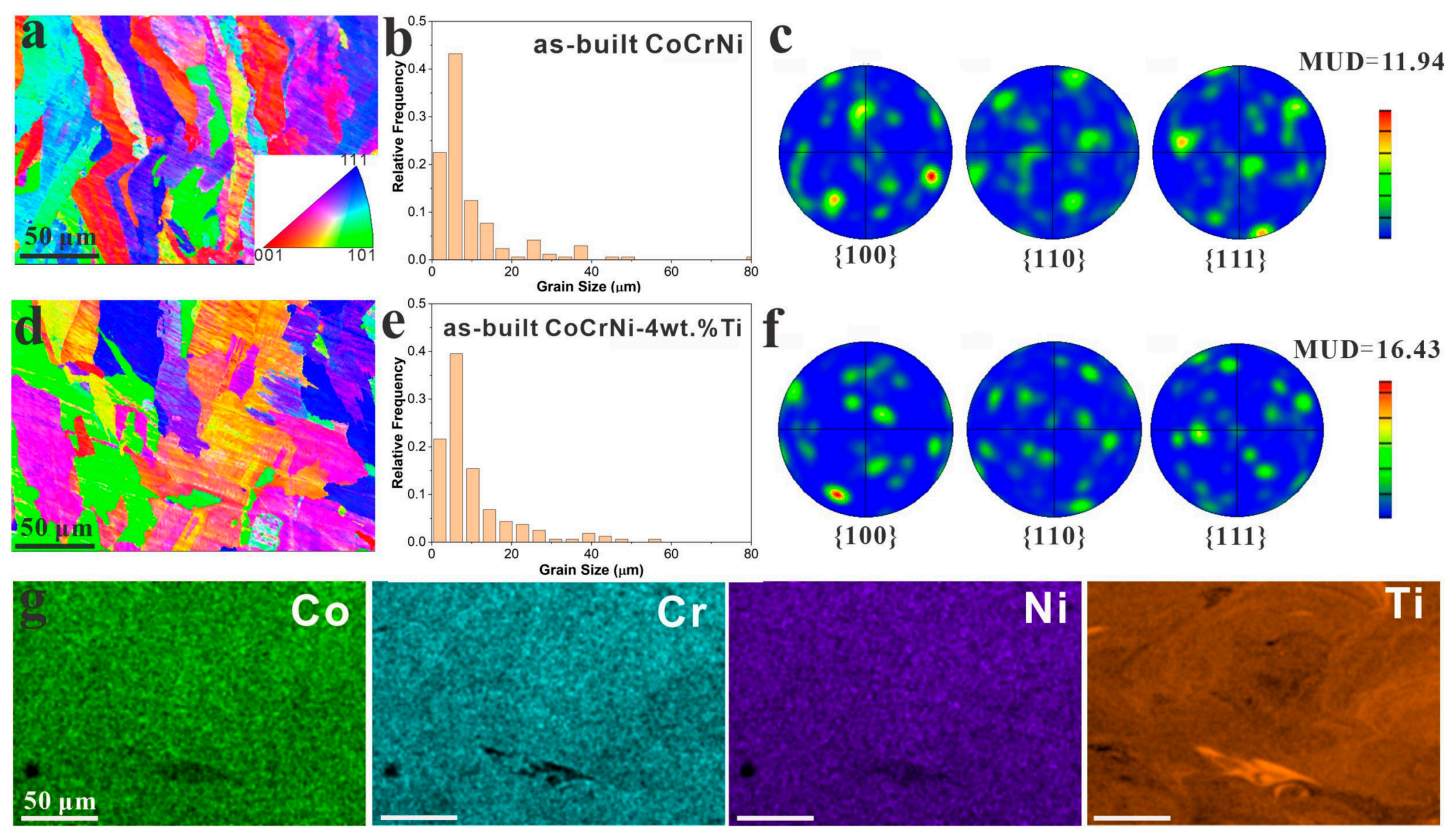
EBSD results of L-PBF (**a**–**c**) CoCrNi alloy in the build direction: (**a**) IPF orientation maps, (**b**) grain boundary distribution maps, (**c**) pole figures. EBSD results of L-PBF (**d**–**g**) CoCrNi-4wt.%Ti alloy in the build direction: (**d**) IPF orientation maps, (**e**) grain boundary distribution maps, (**f**) pole figures, and (**g**) Mapping figure of CoCrNi-4wt.%Ti with different elements.

EBSD was used to investigate the microstructural evolution of grain size and texture of as-built CoCrNi and CoCrNi-4wt%Ti alloys, and the results are presented in Figure 7. Figure 7a,d show the inverse pole figure (IPF) maps, in which color indicates the crystallographic orientation of individual grains. The average grain sizes for CoCrNi and CoCrNi-4wt%Ti were 12.02 and 12.95 μm, respectively. Figure 7c,f show the pole figures of CoCrNi and CoCrNi-4wt%Ti alloys. The maximum multiples of uniform distribution (MUD) values are 11.94 and 16.43 for CoCrNi and CoCrNi-4wt%Ti alloys, respectively. Figure 7g shows the SEM mapping of the L-PBF-ed CoCrNi-4wt%Ti alloy, which exhibits an evenly distributed alloy composition at the microscale. The present results suggest that solute addition alone may not be sufficient to produce obvious grain refinement in L-PBF CoCrNi-4wt.%Ti alloy. The steep thermal gradients and high solidification velocities inherent to L-PBF may limit the effectiveness of solute-driven constitutional supercooling [24]. Table 1 shows the chemical compositions of as-built CoCrNi, CoCrNi-1wt.%Ti, CoCrNi-2wt.%Ti, and CoCrNi-4wt.%Ti alloys.

### 3.2. Mechanical Properties

**Figure 8:**
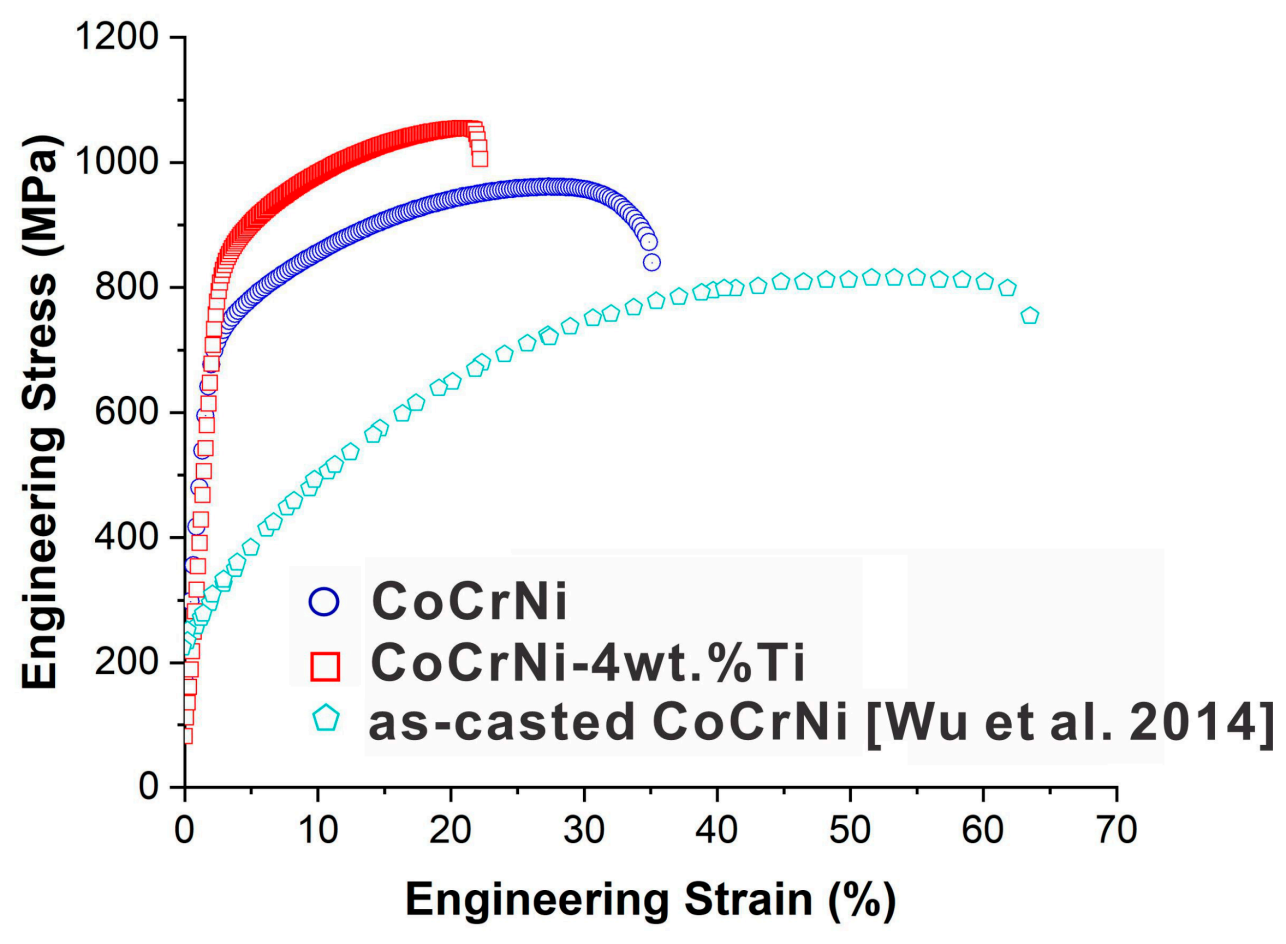
Tensile engineering strain–stress curves of as-bulit CoCrNi and CoCrNi-4wt.%Ti at room temperature compared with as-cast CoCrNi alloy [1].

The representative tensile engineering strain–stress curves of as-built CoCrNi and CoCrNi-4wt.%Ti alloys at room temperature were presented in Figure 8 (Table S2 and Figures S1 and S2 in the Supplementary Materials show the Vickers hardness and tensile test results of L-PBF CoCrNi, CoCrNi-1wt.%Ti, CoCrNi-2wt.%Ti and CoCrNi-4wt.%Ti alloys at different processing parameters). The mechanical properties of as-built CoCrNi, CoCrNi-1wt.%Ti, CoCrNi-2wt.%Ti, and CoCrNi-4wt.%Ti alloys are summarized in Table 2. The as-built CoCrNi alloy exhibited a yield strength of 684.8 MPa, an UTS of 961.1 MPa, and a uniform elongation of 27.8%. The as-built CoCrNi-1wt.%Ti showed a yield strength of 685.2 MPa, a UTS of 949.8 MPa, and a uniform elongation of 26.1%. Interestingly, the CoCrNi-4wt.%Ti reached a yield strength of 777.5 MPa and a UTS of 1054.5 MPa, but the uniform elongation dropped markedly to 20.8%. Compared with the L-PBF CoCrNi alloy, the CoCrNi-4 wt.%Ti alloy showed an increase in yield strength from 684.8 to 777.5 MPa and in ultimate tensile strength from 961.1 to 1054.5 MPa, accompanied by a decrease in uniform elongation from 27.8% to 20.8% (a reduction of seven percentage points, approximately 25% relative). The results show that the enhanced strength at 4 wt.% Ti is not attributed to grain refinement and that a mechanism-based explanation, possibly related to the increased negative enthalpy of mixing, requires further dedicated investigation.

## 4. Conclusions

CoCrNi and CoCrNiTi medium-entropy alloys were prepared using the L-PBF process. The effects of different Ti contents on the microstructure and mechanical properties of the CoCrNi medium-entropy alloy were investigated. The following conclusions were drawn:

(1) The L-PBF CoCrNi-4wt.%Ti alloy processed with a laser power of 275 W and a scanning speed of 1000 mm/s delivered a high yield strength of 777.5 MPa and an ultimate tensile strength of 1054.5 MPa, along with a ductility of 20.8%.

(2) Compared with the L-PBF CoCrNi alloy, the CoCrNi-4 wt.%Ti alloy showed an increase in yield strength from 684.8 to 777.5 MPa and in ultimate tensile strength from 961.1 to 1054.5 MPa, accompanied by a decrease in uniform elongation from 27.8% to 20.8% (a reduction of seven percentage points, approximately 25% relative).

## Figures and Tables

**Table 1:** Chemical compositions of as-built CoCrNi, CoCrNi-1wt.%Ti, CoCrNi-2wt.%Ti and CoCrNi-4wt.%Ti alloys (wt.%).

	**Co**	**Cr**	**Ni**	**Ti**
CoCrNi	35.0 ± 0.3	30.5 ± 0.3	34.5 ± 0.2	-
CoCrNi-1wt.%Ti	34.5 ± 0.4	30.2 ± 0.3	34.2 ± 0.4	1.0 ± 0.2
CoCrNi-2wt.%Ti	34.3 ± 0.4	29.9 ± 0.4	33.9 ± 0.3	1.9 ± 0.3
CoCrNi-4wt.%Ti	33.5 ± 0.3	29.4 ± 0.2	33.2 ± 0.6	3.8 ± 0.8

**Table 2:** Comparison of tensile properties of the as-built CoCrNi, CoCrNi-1wt.%Ti, CoCrNi-2wt.%Ti and CoCrNi-4wt.%Ti alloys.

	**Yield Strength** **(MPa)**	**UTS** **(MPa)**	**Uniform** **Elongation (%)**
CoCrNi	684.8 ± 14.8	961.1± 19.5	27.8 ± 1.8
CoCrNi-1wt.%Ti	685.2 ± 22.3	949.8 ± 20.2	26.1 ± 1.6
CoCrNi-2wt.%Ti	677.9 ± 15.7	938.0 ± 16.9	23.7 ± 2.3
CoCrNi-4wt.%Ti	777.5 ± 18.9	1054.5 ± 19.6	20.8 ± 3.2
As-casted CoCrNi [1]	373	943.8	74.5

## Data Availability

The original findings presented in this study are included in the article; for further inquiries, please contact the corresponding author.

## Supplementary Materials

The following supporting information can be downloaded at: https://www.mdpi.com/article/10.3390/ma19214651/s1, Table S1. Processing parameters for the L-PBF CoCrNi, CoCrNi-1 wt.%Ti, CoCrNi-2 wt.%Ti and CoCrNi-4 wt.%Ti alloys; Figure S1. The Vickers hardness results of L-PBF (a) CoCrNi-0, (b) CoCrNi-1, (c) CoCrNi-2 and (4) CoCrNi-4 alloys at different processing parameters; Figure S2. The tensile tests results of L-PBF (a) CoCrNi-0, (b) CoCrNi-1, (c) CoCrNi-2 and (4) CoCrNi-4 alloys at different processing parameters; Table S2. The yield strength (σ_0.2_), UTS, uniform elongation (UL), hardness and relative density for the L-PBF CoCrNi, CoCrNi-1wt.%Ti, CoCrNi-2wt.%Ti and CoCrNi-4wt.%Ti alloys at different processing conditions.

## Abbreviations

The following abbreviations are used in this manuscript:

L-PBF	Laser Powder Bed Fusion
FCC	Face-Centered Cubic
BCC	Body-Centered Cubic
HCP	Hexagonal close-packed
SEM	Scanning Electron Microscope/Microscopy
XRD	X-Ray Diffraction
EBSD	Electron backscatter diffraction
