# Supplementary material for: Effect of Ti Addition on the Microstructure and Mechanical Properties of L-PBF-Fabricated CoCrNi Medium-Entropy Alloys
Source: Materials (Basel). 2026 Oct 31;19(21):4651. doi: 10.3390/ma19214651 (PMC13765384; doi:10.3390/ma19214651)
Supplement: Supplementary file 1 [file materials-19-04651-s001.zip › materials-4588122-supplementary.pdf]

Supplementary Materials

Effect of Ti Addition on the Microstructure and Mechanical Properties of L-PBF-Fabricated CoCrNi Medium-Entropy Alloys

Zhongqiang Liu [1], Shuya Liang [1], Guan Xu [1] and Siyuan Peng [2,*]

[1] Well Technology Company Limited, Foshan 528225, China; zhqliurp@gmail.com (Z.L.); liangshuya@zhwt.com.cn (S.L.); xg@zhwt.com.cn (G.X.)
[2] School of Materials Science and Engineering, Xiamen University of Technology, Xiamen 361024, China
* Correspondence: PengSY2014@hotmail.com; Tel.: +86-15280200587

Table S1. Processing parameters for the L-PBF CoCrNi, CoCrNi-1 wt.%Ti, CoCrNi-2 wt.%Ti and CoCrNi-4 wt.%Ti alloys.

	Sample IDs	Scanning speed (mm/s)	Laser power (W)	VED（J/mm^3）
CoCrNi-0wt.%Ti	CoCrNi-0-1	1100	250	113.6
	CoCrNi-0-2	1000	250	125
	CoCrNi-0-3	1000	275	137.5
	CoCrNi-0-4	900	250	138.9
	CoCrNi-0-5	950	275	144.7
	CoCrNi-0-6	900	275	152.8
	CoCrNi-0-7	750	250	166.7
	CoCrNi-0-8	1000	350	175
CoCrNi-1wt.%Ti	CoCrNi-1-1	1100	250	113.6
	CoCrNi-1-2	1000	250	125
	CoCrNi-1-3	1000	275	137.5
	CoCrNi-1-4	900	250	138.9
	CoCrNi-1-5	950	275	144.7
	CoCrNi-1-6	900	275	152.8
	CoCrNi-1-7	750	250	166.7
	CoCrNi-1-8	1000	350	175
	CoCrNi-2-1	1100	250	113.6
	CoCrNi-2-2	1000	250	125

CoCrNi-2wt.%Ti	CoCrNi-2-3	1000	275	137.5
	CoCrNi-2-4	900	250	138.9
	CoCrNi-2-5	950	275	144.7
	CoCrNi-2-6	900	275	152.8
	CoCrNi-2-8	1000	350	175
CoCrNi-4wt.%Ti	CoCrNi-4-1	1100	250	113.6
	CoCrNi-4-2	1000	250	125
	CoCrNi-4-3	1000	275	137.5
	CoCrNi-4-4	900	250	138.9
	CoCrNi-4-5	950	275	144.7
	CoCrNi-4-6	900	275	152.8
	CoCrNi-4-7	750	250	166.7

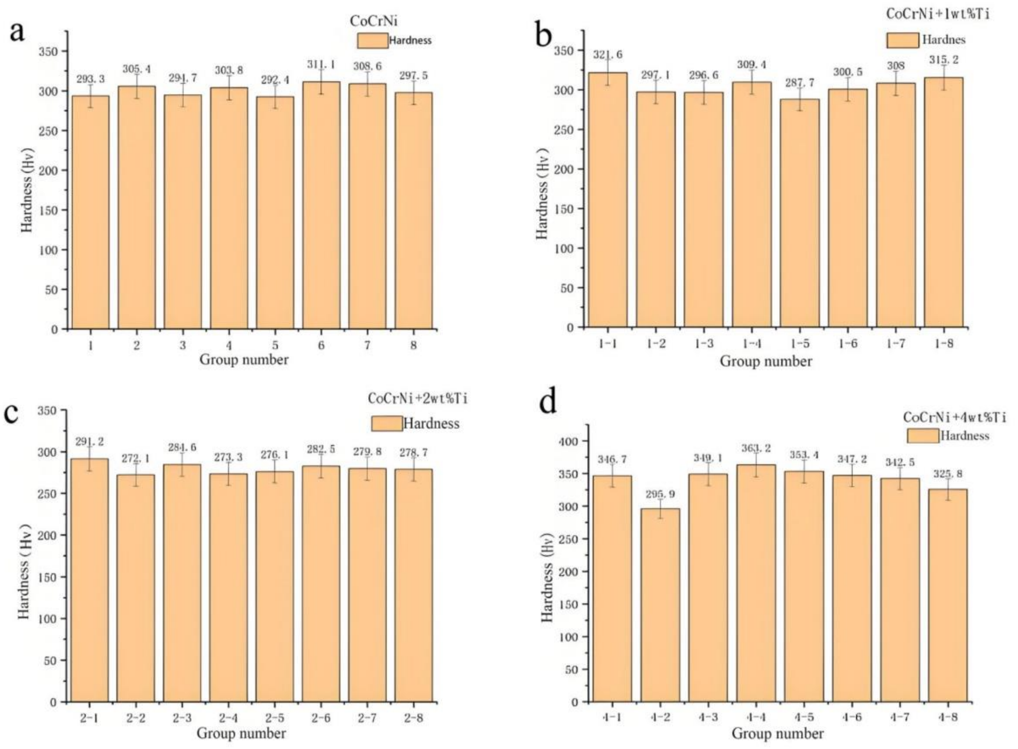

Figure S1. The Vickers hardness results of L-PBF (a) CoCrNi-0, (b) Co*Cr*Ni-*1*, (c) Co*Cr*Ni-*2* and (4) Co*Cr*Ni-4 alloys at different processing parameters.

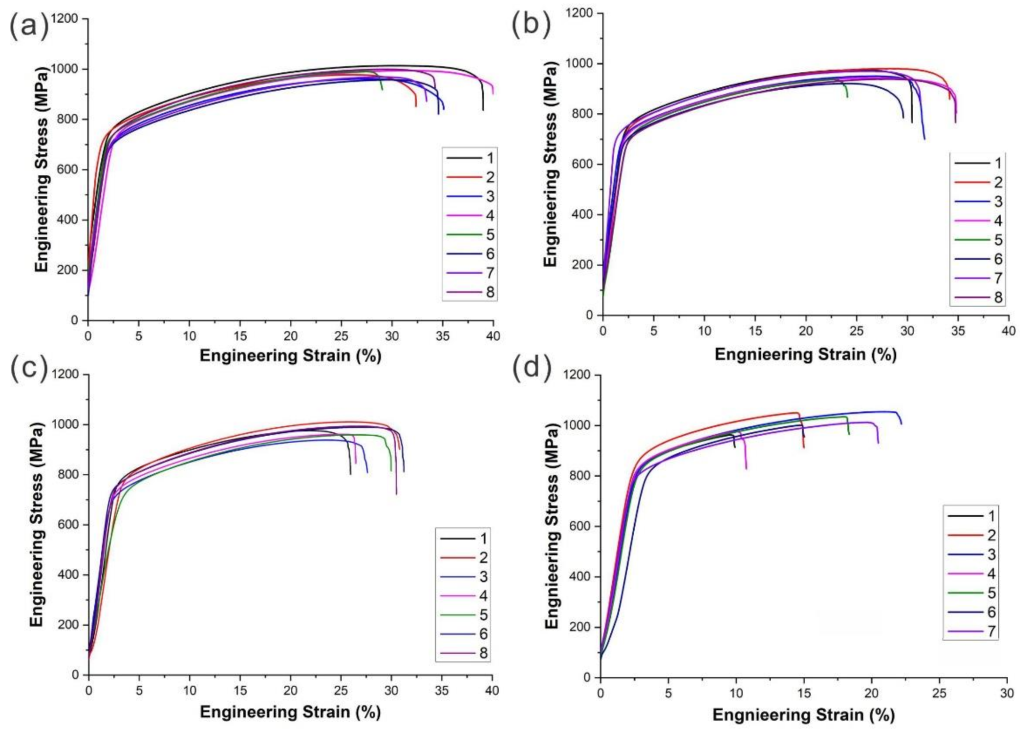

Figure S2. The tensile tests results of L-PBF (a) CoCrNi-0, (b) CoCrNi-*1*, (c) Co*Cr*Ni-*2* and (4) Co*Cr*Ni-4 alloys at different processing parameters.

Table S2. The yield strength ($\sigma_{0.2}$), UTS, uniform elongation (UL), hardness and relative density for the L-PBF CoCrNi, CoCrNi-1wt.%Ti, CoCrNi-2wt.%Ti and CoCrNi-4wt.%Ti alloys at different processing conditions.

Sample IDs	$\sigma_{0.2}$ (MPa)	UTS (MPa)	UL (%)	$HV_{0.2}$	Relative density
CoCrNi-0-1	704.1	1013.8	30.4	293.3 ± 15.2	-
CoCrNi-0-2	663.5	977.6	26.0	305.4 ± 14.3	93.7
CoCrNi-0-3	684.8	961.1	27.8	294.7 ± 14.9	99.9
CoCrNi-0-4	700.6	993.9	31.6	303.8 ± 16.5	93.5
CoCrNi-0-5	700.6	990.3	27.2	292.4 ± 13.8	93.5
CoCrNi-0-6	705.0	999.3	29.4	311.1 ± 15.2	99.8
CoCrNi-0-7	680.9	968.2	28.9	308.6 ± 15.1	88.8
CoCrNi-0-8	703.4	999.3	29.4	297.5 ± 15.0	79.0
CoCrNi-1-1	719.2	975.0	25.1	321.6 ± 14.4	-
CoCrNi-1-2	708.6	980.2	28.2	297.1 ± 14.0	96.5
CoCrNi-1-3	685.2	949.8	26.1	296.6 ± 13.0	95.5
CoCrNi-1-4	689.3	942.2	27.0	309.4 ± 14.1	96.5
CoCrNi-1-5	677.3	936.7	22.6	287.7 ± 12.9	96.7
CoCrNi-1-6	670.4	920.9	24.1	300.5 ± 14.4	95.7

CoCrNi-1-7	701.4	969.8	25.6	308.0 ± 13.2	97.0
CoCrNi-1-8	678.4	938.7	27.9	315.2 ± 14.9	98.0
CoCrNi-2-1	738.7	975.7	22.1	291.2 ± 14.8	94.7
CoCrNi-2-2	729.2	1011.0	25.9	272.1 ± 13.9	94.2
CoCrNi-2-3	677.9	938.0	23.7	284.6 ± 15.1	99.7
CoCrNi-2-4	692.2	961.1	25.2	273.3 ± 13.3	97.2
CoCrNi-2-5	648.2	959.1	26.7	276.1 ± 14.8	99.8
CoCrNi-2-6	715.7	989.6	26.8	282.5 ± 15.5	93.4
CoCrNi-2-8	717.7	993.9	26.8	278.7 ± 14.1	93.8
CoCrNi-4-1	797.5	962.1	9.5	346.7 ± 17.0	97.2
CoCrNi-4-2	806.7	1049.8	14.4	295.9 ± 15.3	98.7
CoCrNi-4-3	777.5	1054.5	20.8	349.1 ± 19.0	99.8
CoCrNi-4-4	791.0	976.0	9.9	363.2 ± 20.3	88.2
CoCrNi-4-5	784.9	1034.8	17.9	353.4 ± 18.4	95.6
CoCrNi-4-6	783.8	1001.0	14.7	347.2 ± 20.3	96.7
CoCrNi-4-7	771.3	1012.6	19.5	342.5 ± 19.3	98.3
